# X-ray magnetic circular dichroism measurements using an X-ray phase retarder on the BM25 A-SpLine beamline at the ESRF

**DOI:** 10.1107/S0909049510005881

**Published:** 2010-03-20

**Authors:** Roberto Boada, María Ángeles Laguna-Marco, Jon Ander Gallastegui, Germán R. Castro, Jesús Chaboy

**Affiliations:** a Instituto de Ciencia de Materiales de Aragón, Consejo Superior de Investigaciones Científicas, CSIC-Universidad de Zaragoza, 50009 Zaragoza, Spain; b Departamento de Física de la Materia Condensada, Universidad de Zaragoza, 50009 Zaragoza, Spain; c Advanced Photon Source, Argonne National Laboratory, Argonne, IL 60439, USA; d Instituto de Ciencia de Materiales de Madrid, Consejo Superior de Investigaciones Científicas, Cantoblanco, 28049 Madrid, Spain; e SpLine, Spanish CRG Beamline at the European Synchrotron Radiation Facility, BP 220, F-38043 Grenoble Cedex, France

**Keywords:** X-ray magnetic circular dichroism, X-ray phase retarder, polarization tunability, magnetic polarization

## Abstract

Circularly polarized X-rays produced by a diamond X-ray phase retarder of thickness 0.5 mm in the Laue transmission configuration have been used for recording X-ray magnetic circular dichroism (XMCD) on the bending-magnet beamline BM25A (SpLine) at the ESRF. Field reversal and helicity reversal techniques have been used to carry out the measurements. The performance of the experimental set-up has been demonstrated by recording XMCD in the energy range from 7 to 11 keV.

## Abstract 2

The experimental set-up at SpLine (BM25A, ESRF) to measure XMCD by using a diamond X-ray phase retarder to obtain circularly polarized X-rays is described.

## 1. Introduction

X-ray magnetic circular dichroism (XMCD), the difference in absorption for left- and right-circularly polarized X-rays by a magnetized sample, constitutes nowadays an outstanding tool for the study of magnetism. In bending-magnet sources at synchrotron radiation facilities a high rate of circularly polarized X-rays, essential to XMCD, can be obtained *before* monochromatization (Ishikawa, 1988 ▶) by selecting the radiation emitted above or below the plane of the electron storage ring (Schütz *et al.*, 1987 ▶). The degree of circular polarization increases with vertical angular aperture but unfortunately at the expense of flux (Duke, 2000 ▶; Stöhr *et al.*, 1998 ▶).

The flux and tunability of polarized X-rays have been improved by using specific insertion devices (IDs) such as elliptical multipole wigglers (Kawata *et al.*, 1988 ▶; Yamamoto *et al.*, 1989 ▶) or helical undulators (Goulon *et al.*, 1995 ▶). However, quick reversal of the photon helicity at these exotic IDs is difficult because this procedure usually involves the rearrangement of magnet arrays.

The development of diffractive X-ray phase retarders (XPRs) (Hirano *et al.*, 1991 ▶, 1994 ▶; Giles *et al.*, 1994 ▶) using transmission geometry has improved the switching ratio of X-ray polarization states. It has also opened the possibility of extending the spin-dependent spectroscopies to every beamline by combining XPR and linearly polarized radiation emitted by undulators (Maruyama, 2001 ▶; Suzuki *et al.*, 1998 ▶; Lang & Srajer, 1995 ▶; Haskel *et al.*, 2001 ▶; Pizzini *et al.*, 1998 ▶) or bending magnets (Bouchenoire *et al.*, 2003 ▶). Moreover, X-ray phase plates can provide well defined polarization without being affected by the finite emittance of the electron beam circulating in the storage ring (Hirano *et al.*, 1991 ▶). Another improvement of XPR usage is given by the helicity modulation technique which combines a fast helicity reversal switching with a lock-in detection, optimizing the signal-to-noise ratio (Hirano *et al.*, 1992 ▶; Suzuki *et al.*, 1998 ▶, 2003 ▶).

In this work we report on the first XMCD measurements performed on the BM25-SpLine beamline at the ESRF. This is a non-XMCD dedicated beamline and the set-up was performed along the duration of an almost standard beam time (ten days) with the handicap that it is a bending-magnet beamline with a large angular divergence. Linearly polarized X-rays were converted into circularly polarized X-rays by using a 0.5 mm-thick diamond X-ray phase retarder working in Laue geometry. Both magnetic field reversal and helicity reversal modes have been used covering an energy range from 7 to 11 keV. The performance of the designed set-up is illustrated by showing XMCD spectra recorded at the Gd and Ho *L*_2_-edges and at the Ge *K*-edge, a nominally non-magnetic atom, in the case of *R*(Fe_1–*x*_Ge_*x*_)_2_ (where *R* is a rare-earth atom) Laves phase compounds.

## 2. X-ray transmission phase plates

The principle of X-ray transmission phase plates is fully described by the dynamical theory of X-ray diffraction that allows for the full interference of electromagnetic waves in a periodic crystal (Batterman & Cole, 1964 ▶). It is known that perfect crystals close to the Bragg condition are birefringent, *i.e.* the π- and σ-polarization components of the electric vector of the incident beam have different phase velocities when they propagate through the crystal that introduces a phase shift δ between them. If the diffraction plane is inclined by an angle ϕ with respect to the electric field of the incident linearly polarized X-ray beam, the circular polarization rate *P*_C_ depends on the phase shift δ in the form *P*_C_ = sin(δ)sin(2ϕ).

To achieve full circularly polarized X-rays both π and σ components must be coherently excited with equal electric field amplitude (ϕ = π/4) and the phase shift between them must be π/2. The latter condition can be fulfilled for each energy by moving out of the Bragg condition by a proper offset angle Δθ since for a chosen phase plate thickness and diffraction configuration this offset at the circular polarization condition depends on energy as ∼*E* ^−4^ (see Hirano *et al.*, 1992 ▶).

## 3. Experimental XMCD set-up

**Figure 1:**
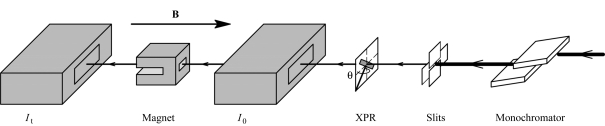
Schematic layout of the set-up for XMCD measurements. This figure is in colour in the electronic version of this paper.

The beamline BM25 (SpLine) (Castro, 1998 ▶) at the ESRF is a bending-magnet beamline split into two different branches each with a horizontal opening angle of 2 mrad, one (branch A) centered at 3.5 mrad and with a critical energy of 9.6 keV, and the other (branch B) centered at 10.5 mrad with a critical energy of 20.6 keV. Branch A is dedicated to X-ray absorption spectroscopy and high-resolution X-ray powder diffraction. A schematic view of the beamline at the XMCD set-up is given in Fig. 1 ▶. The double-crystal monochromator (DCM) used for this branch is a pseudo channel-cut type with two fixed Si (111) crystals moved together by a simple goniometer circle in the (−*n*, +*n*) configuration. The first monochromator crystal is water cooled while the second is kept at room temperature. The second crystal can be finely tilted with respect to the first one in three perpendicular axes. The pitch angle (concentric to the Bragg angle of the crystal) can be regulated during an energy scan in order to keep the transmission of the monochromator optimized during the whole scan, and to reduce the higher-order harmonic content of the beam if necessary. To guarantee a stable beam position and shape on the sample during the energy scan, pre-sample slits (typically of size 1 mm × 1 mm) are placed in front of the sample, and the focusing is tuned to keep the beam just larger than the slits in their position. In this way, even if the beam is moving slightly during the scan, the beam position and shape on the sample are fixed.

**Figure 2:**
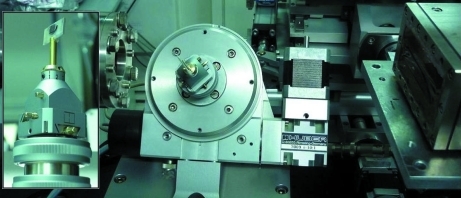
XPR holder and motor stage tilted 45° away from the horizontal plane. This figure is in colour in the electronic version of this paper.

For the XMCD measurements a synthetic 111-diamond plate (Sumitomo Corporation) of thickness 0.5 mm was fixed using beeswax onto a crystal holder plate mounted on a standard pin goniometer (see Fig. 2 ▶). The rotation axis was tilted 45° away from the polarization plane of the incident X-ray beam. We have used the symmetric Laue geometry in which the (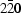) diffraction plane, perpendicular to the crystal surface, is chosen. Two translational motors (*X* and *Z* axes) are used to center the diamond in the beam and a third one rotates the XPR to tune the Bragg condition (θ rotation). In order to obtain circularly polarized X-rays the Bragg peaks are found for each energy point and then the XPR is tuned out of the diffraction condition by adding (subtracting) an offset angle Δθ to obtain left-circularly polarized (right-circularly polarized) light.^1^ A scintillation detector was used to detect the diamond (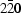) Bragg peaks. The position of this detector was kept fixed for every energy range since its solid acceptance angle is large enough to collect the diffracted beam along a whole energy scan.

**Figure 3:**
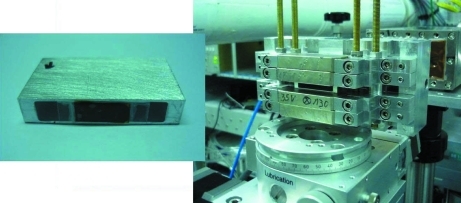
Sample holder and permanent magnet used. This figure is in colour in the electronic version of this paper.

In-house-design magnet for transmission-mode measurements were made using Nd–Fe–B permanent magnets (see Fig. 3 ▶). The magnetic field could be varied by changing the gap between pole pieces. For these experiments the minimum gap, 5 mm, was chosen to obtain a 4.8 kOe magnetic field at the sample position.

## 4. XMCD measurements

XMCD was measured on reference GdFe_2_ and HoFe_2_ samples at the rare-earth *L*_2_-edges. These samples were chosen because their XMCD signals are well characterized and their magnitude and shape allow us to verify that the observed signals are not affected by any spurious signal or derivative effect. Once the performance of the XMCD set-up was verified, the Ge *K*-edge XMCD was recorded on a Gd(Fe_0.9_Ge_0.1_)_2_ sample in which no magnetic role is *a priori* assigned to the Ge atoms.

**Figure 4:**
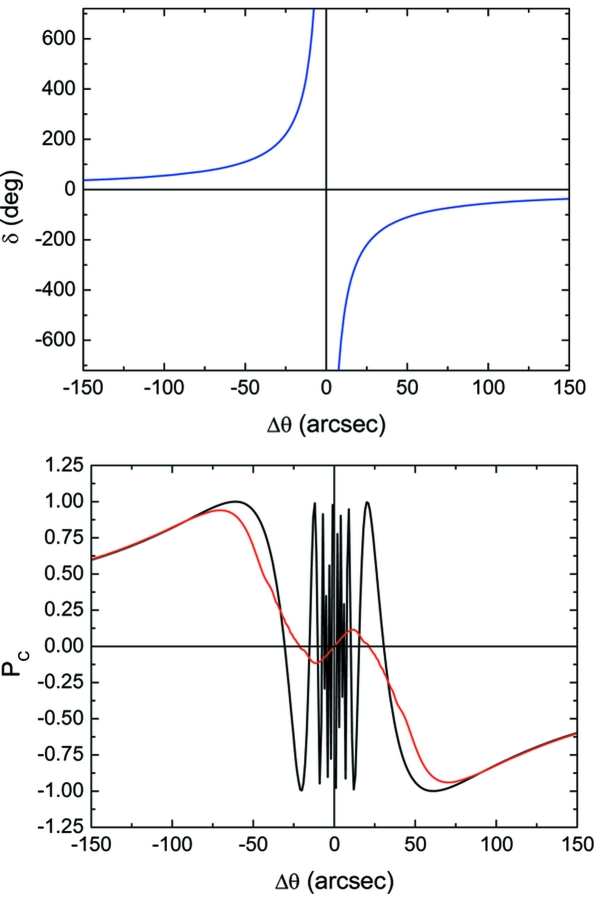
Phase shift (top) and circular polarization rate (bottom) curves as a function of the offset angle. The theoretical curve for *P*_C_ (black line) has been convoluted with a 50 arcsec FWHM Gaussian (red line) to consider the effective divergence of the beam. This figure is in colour in the electronic version of this paper.

**Figure 5:**
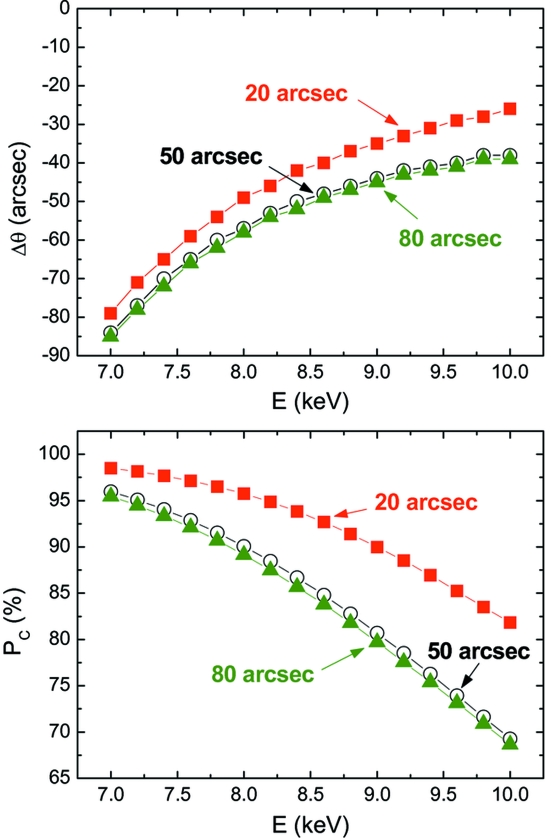
Dependence of the offset angle (top) and circular polarization rate (bottom) on the energy for different FWHM Gaussians used for the convolution: 20 (red solid squares), 50 (black open circles) and 80 arcsec (green solid triangles). This figure is in colour in the electronic version of this paper.

The offset needed to obtain circular polarization conditions was chosen according to the theoretical curves for the circular polarization rate *P*_C_ = sinδ. The theoretical *P*_C_ has been convoluted with a Gaussian function in order to account for the effective divergence of the incident beam, which causes a smearing of the polarization states through the spread of the δ phase shift. However, this effect is minimized by operating the phase plate at large offsets because δ is a slowly varying function of Δθ (see Fig. 4 ▶). The vertical divergence of the beamline has been estimated at about 10 arcsec, while the horizontal divergence has been estimated between 20 and 80 arcsec, depending on the size of the pre-sample slits and the focalization point. The importance of this parameter in selecting a stable offset angle is illustrated in Fig. 5 ▶ where the energy dependence of both the circular polarization rate and the offset angle for different FWHMs of the Gaussian used in the convolution (20, 50 and 80 arcsec) are shown.

For the experiments, XPR tuning was carried out as follows. At each absorption edge energy several Bragg peaks were recorded along the energy range of interest using a scintillation detector. The angle required to move the XPR during each energy scan was obtained by fitting the peak position *versus* energy to an arcsin(*A*/*E*) function, where *A* is a constant. Nevertheless, we have verified that for a small energy window (∼100 eV), as we use to record the XMCD spectra, the error introduced by a linear approximation is negligible. Moreover, we have considered a fixed offset for the whole energy scan. In the case of a 200 eV XMCD scan at the Ho *L*_2_-edge the offset angle varies by ∼2 arcsec. For these reasons the angle needed to move the diamond phase plate during the energy scanning has been varied linearly.

It should be noted that up to now we have considered (see Fig. 5 ▶) the offset Δθ as the small angle out of the Bragg condition that provides the maximum value of the circular polarization rate, *i.e.* the absolute maximum (minimum) of the *P*_C_ curve to obtain right-circularly polarized (left-circularly polarized) light (see bottom panel of Fig. 4 ▶). Actually, this position is not the best choice since it is not stable and small variations could cause undesirable fluctuations in the polarization rate. For this reason it is better to consider a larger value for the offset at the expense of circular polarization rate, to avoid the region close to the diffraction condition. Finally, we have also tested the transmissivity of the XPR for different energy ranges. The results show that in the energy range from the Fe *K*-edge to the Gd *L*_2_-edge (7–8 keV) the diamond slab transmitted ∼30% of the incident beam, in agreement with the theoretical predictions.^2^

**Figure 6:**
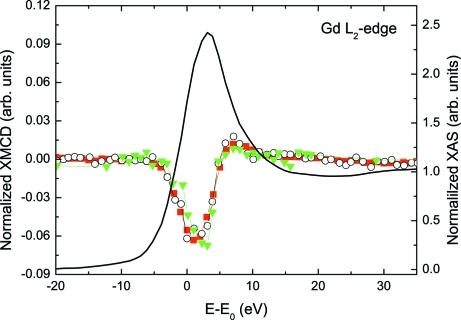
Normalized Gd *L*_2_-edge XMCD spectra recorded on GdFe_2_ using field reversal (green solid triangle), helicity reversal (black open circle) and helicity modulation (red solid square) configurations (see text for details). The normalized XANES spectrum is also shown (black line). This figure is in colour in the electronic version of this paper.

The Gd *L*_2_-edge (7930 eV) XMCD spectra of GdFe_2_ shown in Fig. 6 ▶ were recorded at room temperature and under an applied magnetic field of 4.8 kOe. Both magnetic field and photon helicity reversal techniques were used employing an angular offset equal to 90 arcsec. In all of the cases we have adopted the same convention to display the spectra: the XMCD signal corresponds to the spin-dependent absorption coefficient obtained as the difference of the absorption coefficient μ_c_ = (μ^−^ − μ^+^) for antiparallel, μ^−^, and parallel, μ^+^, orientations of the photon helicity and the magnetic field applied to the sample. A comparison of the results obtained using both methods is reported in Fig. 6 ▶, where the XMCD signals have been normalized to the absorption jump and corrected by the estimated circular polarization rate (∼0.8). Both measuring methods yield the same spectral shape characterized by a negative peak, ∼5 eV wide, centered at ∼1 eV above the edge, in agreement with previous results (Laguna-Marco *et al.*, 2005 ▶, 2008*a* ▶,*b* ▶). For further verification of the reliability of these results, the same specimen was measured in transmission mode at the undulator beamline BL39XU at SPring-8 (proposal No. 2008A1051). The helicity modulation technique was used with a 0.7 mm-thick diamond phase retarder. The good agreement between both measurements, reported in Fig. 6 ▶, points out the high performance of the SpLine set-up.

**Figure 7:**
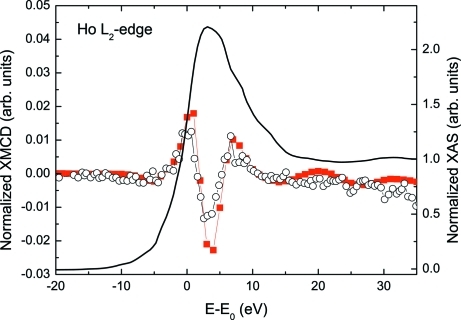
Comparison of normalized Ho *L*_2_-edge XMCD signals recorded on HoFe_2_ by using the helicity reversal (black open circle) and helicity modulation techniques (red solid square). The normalized XANES spectrum is also shown (black line). This figure is in colour in the electronic version of this paper.

Similar results have been obtained in the case of the Ho *L*_2_-edge (8918 eV) XMCD of HoFe_2_. The XMCD signal was recorded by using the helicity reversal method with an offset of 61 arcsec that corresponds to an estimated circular polarization rate higher than 0.7. As shown in Fig. 7 ▶, the XMCD signals recorded on the same specimen at both SpLine and BL39XU show a remarkable agreement: the Ho *L*_2_-edge XMCD exhibits a positive peak at ∼1 eV above the edge, a negative peak at ∼4 eV and another positive peak centered at ∼7 eV above the edge. The measurements at BL39XU were performed under the same experimental conditions as mentioned for the Gd *L*_2_-edge case. It should be noted, however, that the amplitude of the spectrum recorded in our experimental set-up is slightly smaller than that recorded at BL39XU. This effect might be addressed to the loss of the correct XPR position during the measurement since no encoder was used for the rotation. However, this hypothesis can be discarded because the diffraction peaks measured after the XMCD measurement are close to the expected values. Therefore, we tentatively assign this reduction to the different harmonic rejection method used (second crystal of the DCM detuning at SpLine and flat Rh-coated mirror after the XPR at BL39XU) since the high-order harmonics contamination can distort the XMCD measurement.

Finally, in order to test the set-up at high energy, *i.e.* when the transmission through the diamond is higher and hence the circular polarization is lower, we have recorded the XMCD at the Ge *K*-edge (11103 eV). To this end we have considered the case of the Gd(Fe_0.9_Ge_0.1_)_2_ Laves phase compound in which magnetic Fe atoms have been substituted by non-magnetic Ge atoms. Recent works have shown the existence of non-zero XMCD signals at the *K*-edge of atoms like Ge or Ga (Inada *et al.*, 2005 ▶; Haskel *et al.*, 2007 ▶; Chaboy *et al.*, 2009 ▶). These results illustrate the importance of XMCD in determining the exact nature of the induced magnetic moments in traditionally non-magnetic atoms owing to the interplay of the hybridization and of the modification of the electronic structure.

**Figure 8:**
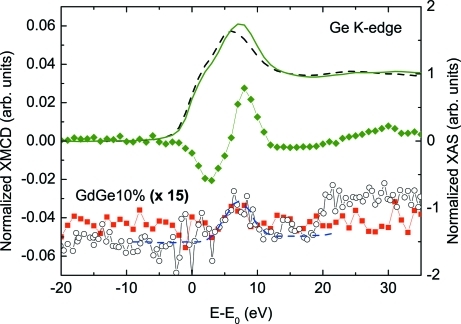
Comparison of normalized XMCD signals of Gd(Fe_0.9_Ge_0.1_)_2_ recorded at the Ge *K*-edge using helicity reversal (black open circles) and helicity modulation techniques (red solid squares). The dashed (blue) line is a guide to the eye. The XMCD signal of Ho(Fe_0.9_Ge_0.1_)_2_ at 5 K and 50 kOe is also shown (green solid diamonds). For the sake of completion the normalized XANES spectra is also shown for Gd(Fe_0.9_Ge_0.1_)_2_ (black dashed line) and Ho(Fe_0.9_Ge_0.1_)_2_ (green line). This figure is in colour in the electronic version of this paper.

We have recorded the Ge *K*-edge XMCD by using an angular offset of 34 arcsec for which the theoretical estimate yields a circular polarization rate of ∼0.6.^3^ The results are reported in Fig. 8 ▶ where the XMCD signal measured in the set-up developed at SpLine is compared with that recorded at the XMCD-dedicated station of BL39XU at SPring-8. At BL39XU the helicity modulation technique with a 1.4 mm-thick diamond XPR was used to record the XMCD signal in transmission mode (proposal No. 2008B1753). In both cases the XMCD spectra show a spectral feature of positive sign centered at ∼7 eV above the edge, whose amplitude is only about 0.1% of the absorption jump.^4^ For the sake of comparison we also show, in Fig. 8 ▶, the XMCD signal of Ho(Fe_0.9_Ge_0.1_)_2_ at the Ge *K*-edge recorded at 5 K and under a magnetic field of 50 kOe. In this case, for the Ho Laves phase compound, the Ge magnetic polarization is larger and the XMCD signal is clearer than in the Gd compound case. We have measured the Gd compound instead of the Ho compound because the goal was to illustrate the performance of the XMCD set-up at SpLine at the detection limit observed at BL39XU. These results constitute the first evidence of the magnetic polarization of the Ge atoms in the diluted *R*(Fe_1–*x*_Ge_*x*_)_2_ Laves phase materials.

## 5. Summary and conclusions

We have reported the first XMCD measurements performed on the bending-magnet BM25 A-SpLine beamline at the ESRF. Linearly polarized X-rays were converted into circularly polarized X-rays by using a 0.5 mm-thick diamond X-ray phase retarder working in the Laue geometry, and the results are in good agreement with the prediction for the polarization rate. Both magnetic field reversal and helicity reversal modes have been used to record XMCD in an energy range from 7 to 11 keV. The quality of the dichroic signals obtained is comparable with that recorded at the XMCD-dedicated beamline BL39XU at SPring-8.

## Footnotes

1 We consider the atomic convention for the left- and right-handed sense of circularly polarized light (viewed from the source).

2 Theoretical predictions give a transmission of ∼20% and ∼35% at 7 and 8 keV, respectively, for a diamond slab in the Laue (220) configuration.

3 We are conscious that the circular polarization rate obtained with a 111-diamond of thickness 0.5 mm in the Laue(220) symmetric geometry is not optimum at the Ge *K*-edge energies, it being more convenient to use a thicker diamond phase plate to obtain a higher degree of circular polarization. However, it serves our purpose of checking the circular polarization rate achieved at higher energy in our experimental set-up.

4 The Ge *K*-edge signal recorded at SpLine shows a poorer signal-to-noise ratio than that recorded at BL39XU where a 1.4 mm-thick XPR, providing a higher circular polarization rate than the 0.5 mm-thick XPR of SpLine, was used.
